# Ernährungsempfehlungen für Menschen mit Diabetes (Update 2023)

**DOI:** 10.1007/s00508-023-02170-y

**Published:** 2023-04-20

**Authors:** Carmen Klammer, Karin Schindler, Rita Bugl, Dagmar Plazek, Miriam Vötter, Tanja Kirchner, Claudia Martino, Jasmin Klammer-Martin, Johanna Brix, Sabine Dämon, Friedrich Hoppichler, Alexandra Kautzky-Willer, Renate Kruschitz, Hermann Toplak, Martin Clodi, Bernhard Ludvik

**Affiliations:** 1 Abteilung für Innere Medizin, Konventhospital der Barmherzigen Brüder Linz, Linz, Österreich; 2 ICMR – Institute of Cardiovascular and Metabolic Research, Johannes Kepler Universität Linz, Altenberger Straße 69, 4040 Linz, Österreich; 3 Bundesministerium für Soziales, Gesundheit, Pflege und Konsumentenschutz, Wien, Österreich; 4 Klinische Abteilung für Endokrinologie und Stoffwechsel, Universitätsklinik für Innere Medizin III, Medizinische Universität Wien, Wien, Österreich; 5 Wiener Gesundheitsverband Klinik Ottakring, Wien, Österreich; 6 Landesklinikum Melk, Melk, Österreich; 7 Bezirkskrankenhaus Schwaz, Schwaz, Österreich; 8 Österreichische Gesundheitskasse Mein Peterhof Baden, Baden, Österreich; 9 Österreichische Gesundheitskasse Mein Gesundheitszentrum Floridsdorf, Wien, Österreich; 10 Ernährungsberatung Klammer, Enns, Österreich; 11 Medizinische Abteilung mit Diabetologie, Endokrinologie und Nephrologie, Klinik Landstraße, Wien, Österreich; 12 Special Institute for Preventive Cardiology and Nutrition, SIPCAN – Initiative für ein gesundes Leben, Elsbethen/Salzburg, Österreich; 13 Abteilung für Innere Medizin, Krankenhaus der Barmherzigen Brüder Salzburg, Salzburg, Österreich; 14 Gender Medicine Unit, Klinische Abteilung für Endokrinologie und Stoffwechsel, Universitätsklinik für Innere Medizin III, Medizinische Universität Wien, Wien, Österreich; 15 Abteilung für Innere Medizin, Krankenhaus der Elisabethinen, Klagenfurt, Österreich; 16 Klinische Abteilung für Endokrinologie und Diabetologie, Universitätsklinik für Innere Medizin, Medizinische Universität Graz, Graz, Österreich

**Keywords:** Körpergewicht, Kohlenhydratzufuhr, Proteinzufuhr, Fettzufuhr, Mikronährstoffe, Body weight, Diatery carbohydrates, Diatery protein, Diatery fats, Micronutrients

## Abstract

Je nach Diabetesform und -therapie sollen alle Menschen mit Diabetes eine individuelle ernährungsmedizinische Beratung und Schulung durch Fachpersonal erhalten. Im Vordergrund sollte eine patientinnen- und patientenzentrierte, individualisierte Beratung stehen, angepasst an die jeweiligen Bedürfnisse und Lebensumstände der Menschen mit Diabetes. Neben der Unterstützung zur Umsetzung einer ausgewogenen Ernährung gilt es, gemeinsam mit Patient:innen individuelle Stoffwechselziele und Gewichtsziele zu definieren, um mithilfe der Ernährung den Krankheitsverlauf positiv zu beeinflussen und mögliche Spätfolgen zu vermeiden. Dabei sollten vor allem praxisbezogene Empfehlungen unter Berücksichtigung der persönlichen Nahrungsmittel-Präferenzen ausgesprochen werden und Hilfsmittel zur Planung von geeigneten Portionsgrößen und der geeigneten Mahlzeitenzusammenstellung zum Einsatz kommen. Entsprechend aktueller internationaler und nationaler Standards sollen Menschen mit Diabetes im Diabetes-Selbstmanagement unterstützt werden (DSMES) und erlernen, die postprandiale Reaktion auf Speisen und Getränke besser einschätzen und durch die geeignete Lebensmittel- und Getränkeauswahl positiv beeinflussen zu können. Alle Menschen mit Diabetes sollten regelmäßig, je nach individuellem Bedarf, die Möglichkeit haben, eine ernährungstherapeutische Beratung oder Schulung in Anspruch nehmen zu können.Diese Praxisempfehlung stellt eine Zusammenfassung der aktuellen Literatur zu ernährungsrelevanten Aspekten bei Diabetes dar.

## Abstract (en)

All patients with diabetes require individual and personalized nutritional consultation with professionals. The patient’s needs should be the primary focus of the dietary therapy, taking their lifestyle and the type of diabetes into consideration. With the recommendations to the patient’s diet, there need to be specific metabolic goals to reduce the disease’s progression and to avoid long term health effects. Therefore, practical guidelines such as portion size and meal planning tips should be the main focus.According to the latest national and international standards, patients suffering from diabetes should have access to nutrition consulting and nutritional training. During consultation they can be supported on- how to manage their health condition and choosing food and beverage to improve their health.These practical recommendations sum up the latest literature on nutritional aspects of diabetes treatment.

## 1. Makronährstoffe

Die Makronährstoffverteilung sollte grundsätzlich individuell basierend auf den Essgewohnheiten, Nahrungspräferenzen, körperlicher Aktivität und den Stoffwechselzielen erfolgen. Es gibt Hinweise, dass es für Diabetes Betroffene keine ideale prozentuale Verteilung der Makronährstoffe gibt, die allgemein gültig ist [1, 2] – auf diese wird jeweils in den folgenden Kapiteln eingegangen. Weiters sollte ein Augenmerk auf die Gesamtenergiezufuhr gelegt werden, um das Ziel einer eventuellen notwendigen Gewichtsabnahme zu erreichen. Die Schlüsselstrategie in der Beratung liegt im Erreichen der glykämischen Ziele, in der Bewertung der Nährstoffaufnahme sowie in der Optimierung der Auswahl von Lebensmitteln und Etablierung ausreichend körperlicher Aktivität im Alltag.

### 1.1 Kohlenhydrate

Menschen mit Typ 1‑ und Typ 2‑Diabetes können zwischen 45 und 55 % des Gesamtenergiebedarfs in Form von Kohlenhydraten aufnehmen. Dies entspricht bei einer Energiezufuhr von 2000 kcal, im Mittel ca. 250 g Kohlenhydrate täglich [3]. Bei der Auswahl von kohlenhydrathaltigen Lebensmitteln steht die Qualität vor der Quantität, daher sollten nährstoffreiche Kohlenhydratquellen, die ballaststoffreich und möglichst wenig verarbeitet sind, bevorzugt werden. Bei insulinabhängigen Patient:innen ist es von besonderer Bedeutung, den Zusammenhang zwischen Kohlenhydraten und Insulin verständlich zu machen. Dies soll im Rahmen einer intensiven Schulung zum Kohlenhydratgehalt von Speisen in Gramm an Kohlenhydraten bzw. in Kohlenhydrateinheiten (KE) je nach Therapieform erfolgen.

#### 1.1.1 Glykämischer Index

Bei der Auswahl von kohlenhydratreichen Lebensmitteln ist neben dem Ballast-stoffgehalt auch der Glykämische Index (GI) bzw. die Glykämische Last (GL) zu beachten. Der Einfluss von Nahrungskohlenhydraten auf die glykämische Antwort hängt von verschiedenen Faktoren wie aufgenommener Menge, Art und zellulärer Struktur, thermischer und/oder mechanischer Verarbeitung sowie gleichzeitigem Verzehr anderer Makronährstoffe ab [4]. Darüber hinaus wird die glykämische Antwort auf Nahrungsmittel auch von der Nüchternblutglukosekonzentration und dem Ausmaß der Insulinresistenz beeinflusst.

Der GI ist eine Maßzahl für die Wirksamkeit verschiedener Lebensmittel auf die Blutglukose. Seine Bestimmung erfolgt, indem die Blutzuckerkurve nach Aufnahme von 50 g Kohlenhydraten aus einem Testlebensmittel über 2 h verfolgt wird. Diese Kurve wird zu jener in Beziehung gesetzt, welche sich aus dem Konsum von 50 g Kohlenhydraten in Form von Weißbrot oder Glukose ergibt. Der GI wird in Prozent in Bezug zum Referenzlebensmittel angegeben. Daher bedeutet ein GI = 70, dass die Blutzuckerwirksamkeit des untersuchten Lebensmittels 70 % der von 50 g Weißbrot bzw. Glukose beträgt (die Fläche der Blutzuckerkurve ist um 30 % geringer als die von Weißbrot bzw. Glukose). Die Auswirkungen eines Lebensmittels auf den Blutglukose- und Insulinspiegel hängen sowohl von der Menge der verzehrten Kohlenhydrate als auch vom GI ab. Die alleinige Betrachtung des GI hat den Nachteil, dass er sich definitionsgemäß auf 50 g Kohlenhydrate bezieht, welche nur vereinzelt praxisbezogene Verzehrgewohnheiten widerspiegelt. Folglich entsprechen 50 g Kohlenhydrate aus Karotten einer Menge von 650 g, sodass der Verzehr einer üblichen Portion zwischen 100–150 g trotz des höheren GI geringe Auswirkungen auf den Blutglukosespiegel hat. Die Verzehrgewohnheiten werden im Konzept der GL berücksichtigt. Die GL errechnet sich aus dem Produkt der verwertbaren Kohlenhydratmenge pro Portion und dem GI [5]. Eine Ernährungsweise mit niedrigem GI/GL kann sich demnach positiv auf den Blutglukosespiegel auswirken, aber wahrscheinlich genau deshalb, weil vorrangig minimal verarbeitete und ballaststoffreiche Lebensmittel, wie Vollkorn, Obst und Gemüse sowie hochwertige Proteinquellen verzehrt werden [6]. Eine Ernährungsweise mit niedrigem GI/GL basiert auf minimal verarbeiteten und ballaststoffreichen Lebensmitteln, wie Vollkornprodukte, Obst und Gemüse und wirkt somit positiv auf den Blutglukosespiegel [6].

### 1.2 Ballaststoffe

Der häufig beobachtete ungünstige Effekt einer stärkereichen Ernährung auf den Triglyzerid-Plasmaspiegel [7] kann vermieden werden, wenn die verzehrten kohlenhydratreichen Lebensmittel gleichzeitig ballaststoffreich sind. Daher sind Vollkorngetreideprodukte Weißmehlprodukten vorzuziehen. Eine tägliche Ballaststoffaufnahme von 30 g/d wird empfohlen. Dies kann durch den Konsum von Getreide bzw. Vollkorngetreide, Hülsenfrüchten, Nüssen, Samen, Gemüse und moderaten Mengen Obst erreicht werden. Höhere Aufnahmen waren mit größeren positiven Effekten assoziiert [8]. Bei einer höheren Aufnahme bzw. bei einer Steigerung der Ballaststoffaufnahme ist zu berücksichtigen, dass es zu Obstipation oder Flatulenzen kommen kann, zudem ist auch auf eine passende Flüssigkeitszufuhr zu achten [9]. In der Literatur werden positive Effekte auf die Blutglukose und Insulinresistenz sowie eine geringere Mortalität beschrieben verglichen mit einer niedrigeren Ballaststoffaufnahme [10]. Die Hälfte der Ballaststoffe sollte in Form von löslichen Ballaststoffen aufgenommen werden (z. B. Pektine, Inulin, Psyllium). Diese finden sich vor allem in Gemüse und Obst. Der Verzehr von Ballaststoffen in Form von natürlichen Lebensmitteln ist dem von ballaststoffreichen Nahrungsergänzungsmitteln vorzuziehen, da Lebensmittel auch Mikronährstoffe liefern [11, 12].

#### 1.2.1 Insulinresistenz und Beta-Glucan

Der im Hafer enthaltene lösliche Ballaststoff β‑Glucan zeigt in Studien positive Auswirkungen auf das Glukose- und Lipidprofil bei Personen mit Diabetes mellitus Typ 2 (T2DM). Die Aufnahme von 3 g β‑Glucan pro Tag, enthalten in rund 60 g Hafer, kann bei positivem Wirkungsprofil für Personen mit T2DM empfohlen werden. Für jene mit Diabetes mellitus Typ 1 (T1DM) fehlen noch entsprechende Daten [13, 14].

In Studien waren natürliche Haferflocken dem Extrakt von β‑Glucan überlegen [15, 16]. Zweitägige, hypokalorische Hafer- bzw. Ballaststofftage sind besonders im stationären Umfeld zur Therapie einer ausgeprägten Insulinresistenz zu empfehlen. In einer Pilotstudie konnte bei zweitägigen Hafertagen mit 1100 kcal aus Haferbrei eine Reduktion der mittleren Insulindosis von 47 % erreicht werden [17]. In vitro konnte eine hemmende Wirkung auf die SGLT1-Rezeptoren, die GLUT2-Transporter, die Dipeptidylpeptidase 4 (DPP4) und die Alpha-Glukosidase beobachtet werden [17–21]. Zudem bindet β‑Glucan Gallensäuren im Darm und entzieht sich somit dem enterohepatischen Kreislauf, wodurch der Cholesterinspiegel gesenkt werden kann [22, 23]. Hafertage sollten immer unter ärztlicher und ernährungstherapeutischer Begleitung stattfinden und im Optimalfall stationär durchgeführt werden.

### 1.3 Fett

Der Anteil der täglich aufgenommenen Energie aus Fetten sollte 35 % der Gesamtenergie nicht überschreiten. Daraus berechnen sich bei einer täglichen Energiezufuhr von 2000 kcal ca. 60–80 g Gesamtfett pro Tag. Darüber hinaus ist es von besonderer Bedeutung, die Qualität des aufgenommenen Fettes zu beachten bzw. zu modifizieren.

Maximal 10 % der täglichen Gesamtenergiezufuhr dürfen, wie bei gesunden Erwachsenen, in Form von gesättigten Fettsäuren und Transfettsäuren aufgenommen werden. Transfettsäuren entstehen bei der Hydrogenierung pflanzlicher Öle bzw. im Pansen von Wiederkäuern. Gesättigte Fettsäuren sind vor allem in tierischen Lebensmitteln und streichfähigen Fetten zu finden. Sie sind der diätetische Faktor mit den größtmöglichen Auswirkungen auf den Serum-Cholesterinspiegel.

Die Aufnahme mehrfach ungesättigter Fettsäuren (PUFA) sollte ebenfalls 10 % der täglichen Gesamtenergieaufnahme nicht überschreiten.

Es gibt Hinweise, dass der Austausch von gesättigten Fettsäuren durch einfach oder mehrfach ungesättigte Fettsäuren einen protektiven Effekt in der Prävention der koronaren Herzkrankheit (KHK) hat. Der Austausch der gesättigten Fettsäuren mit Kohlenhydraten senkt das Risiko hingegen nicht. Eine Modifikation der Lebensmittelauswahl und der Ernährungsgewohnheiten kann auch eine deutliche Verbesserung im Sinne der sekundären Prävention der KHK bewirken [24]. In Bezug auf die Diabetesprävention und den Einfluss einer optimalen Fettaufnahme bzw. Fettsäurezusammensetzung auf Cholesterinwerte im Blut und des kardiovaskulären Risikos werden weitere Studien benötigt [25].

Fischöl-Supplemente können bei Menschen mit T2DM den Triglyzerid-Spiegel senken [1]. Eine generelle Supplementierung mit Fischölen bei Personen mit Diabetes ohne ein kardiovaskuläres Risiko kann aber derzeit nicht empfohlen werden [26]. Nahrungsmittel, die reich an langkettigen Omega‑3 Fettsäuren sind, wie beispielsweise fetter Fisch, Nüsse oder Samen, sind zur Prävention und Behandlung von kardiovaskulären Erkrankungen vorzuziehen [27]. Vegane „Fischöl“-Supplemente, die aus Algen gewonnen werden, sind ökologisch wesentlich besser.

Eine tägliche Aufnahme von 5 g Transfettsäuren und mehr, erhöht das kardio-vaskuläre Risiko um 25 % [28]. In verschiedenen Studien wurde ein LDL-Cholesterin-steigender und HDL-Cholesterin-senkender Effekt beobachtet. Die Frage, ob ein höherer Konsum von Transfettsäuren mit einem höheren Diabetesrisiko verbunden ist, kann derzeit nicht endgültig beantwortet werden. Die Minimierung der Aufnahme von Transfettsäuren erscheint jedenfalls angezeigt. In Europa ist ihr quantitativer Anteil in Margarinen aufgrund verbesserter Produktionsbedingungen jedoch vernachlässigbar. Zu berücksichtigen sind zudem andere mögliche Quellen für Transfettsäuren wie Fastfood-Produkte und fettreiche Backwaren. Natürliche Transfette, die beispielsweise in Milchprodukten oder Rindfleisch enthalten sind, wurden in epidemiologischen Studien mit einem abgesenkten Diabetesrisiko in Zusammenhang gebracht werden [29].

### 1.4 Eiweiß

Der Anteil der täglichen Proteinaufnahme an der Gesamtenergiezufuhr kann bei Personen unter 65 Jahren und ohne Anzeichen einer Nephropathie 10–20 Energieprozent (E%), also 0,8–1,3 g/kg/Körpergewicht (KG), betragen. Älteren und geriatrischen Menschen (> 65 Jahre) wird jedoch eine höhere Eiweißzufuhr von 1 g/kg/KG (15–20 E%) am Tag empfohlen, um eine Mangelernährung zu vermeiden [3, 30, 31]. Während einer energiereduzierten Diät zur Gewichtsabnahme ist darauf zu achten, dass die adäquate Proteinaufnahme sichergestellt ist.

Inwiefern eine höhere Proteinaufnahme (> 20 % der täglichen Energieaufnahme) sich langfristig auf die Entwicklung einer Nephropathie auswirkt, ist noch nicht endgültig geklärt. Die Proteinaufnahme in den üblichen Mengen (≈ 1 g/kg/KG) erscheint sicher [32, 33]. Der Blutglukosespiegel wird durch die Proteinaufnahme nicht erhöht, allerdings stimuliert Nahrungsprotein die Insulinsekretion [34]. Eine Beschränkung der Proteinaufnahme, um die Entwicklung einer Albuminurie und das Fortschreiten einer (diabetischen) Nephropathie zu reduzieren, wurde in der Vergangenheit empfohlen – die Ergebnisse vieler klinischen Studien, Metaanalysen und Reviews sind allerdings kontrovers [2, 30]. In einer Metaanalyse konnte eine Proteinrestriktion auf

0,6–0,8 g/kg/KG keine Verbesserung der Nierenfunktion zeigen [35]. Extreme Restriktionen auf 0,3–0,4 g/kg/KG zeigten in einer Cochran-Analyse eine gering signifikante Reduktion der Niereninsuffizienz [36, 37]. Solche derartigen Einschränkungen sind jedoch in der Praxis schwer durchführbar und bergen ein erhöhtes Risiko einer Mangelernährung, welche wiederum mit einer erhöhten Mortalität in Zusammenhang stehen [38–41]. Eine Proteinaufnahme gemäß der normalen Empfehlungen bei Personen mit diabetischer Nephropathie und einer glomerulären Filtrationsrate zwischen 60 ml/min und 45 ml/min konnte nicht mit einer rascheren Verschlechterung der Nierenfunktion in Zusammenhang gebracht werden [30]. Übereinstimmend mit dem Konsensuspaper der Amerikanischen Diabetes-gesellschaft wird eine eingeschränkte Eiweißzufuhr bei Niereninsuffizienz nicht empfohlen [2].

In den letzten Jahren wurde der Einfluss einer proteinreichen, kohlenhydratarmen Diät auf das Ausmaß der Gewichtsabnahme sehr kontrovers diskutiert. Energiereduzierte, proteinreiche vs. kohlenhydratreiche Diäten über sechs Monate, resultierten bei Gesunden in einer signifikant besseren Gewichtsabnahme [42]. Eine randomisierte Langzeitstudie untersuchte den Einfluss einer proteinreichen Ernährung (30 E% Protein) versus eine kohlenhydratreiche Ernährung (15 E% Protein) auf das Körpergewicht von Proband:innen mit T2DM. Die proteinreiche Ernährung hatte keinen signifikant besseren Einfluss auf das Körpergewicht und den Bauchumfang [32]. Es ist anzumerken, dass ein Proteinanteil von 30 % an der Gesamtenergie-aufnahme nicht praktikabel zu sein scheint – im Mittel lag die Proteinaufnahme zwischen 20 und 21 E% [32]. Proteinreiche Diäten favorisieren jedoch in der Regel eine hohe Aufnahme von Cholesterin und gesättigten Fettsäuren – der Obst- und Gemüsekonsum wird stark eingeschränkt – sie müssen daher im Hinblick auf die Prävention einer Arteriosklerose kritisch betrachtet werden.

### 1.5 Mikronährstoffe und Spurenelemente

Die ausreichende Aufnahme von Mikronährstoffen (Vitaminen und Spurenelementen) ist ein wichtiger Faktor zur Erhaltung der Gesundheit von Menschen mit Typ 1‑ und Typ 2‑Diabetes. Die empfohlene tägliche Zufuhr unterscheidet sich nicht von der für gesunde Erwachsene. Lebensmittel, die reich an Vitaminen und Spurenelementen sind, sollten daher bevorzugt werden.

Vor allem Schwangeren, Stillenden, Patienten nach metabolisch-bariatrischen Operationen, älteren Patient:innen und solchen, die eine energiereduzierte Ernährungsweise einhalten, kann eine Supplementierung mit einem Multivitaminpräparat empfohlen werden. Eine ständige Supplementierung von Mikronährstoffen in Dosierungen über der empfohlenen Tagesmaximaldosis ist besonders beim Fehlen von klinischen bzw. laborchemischen Mangelzuständen abzulehnen.

#### 1.5.1 Nahrungsergänzungsmittel

##### Vitamin B12

Eine Langzeit-Metformingabe kann mit einem erniedrigten Vitamin-B12-Spiegel assoziiert sein. Die Einnahme von Protonenpumpenhemmern kann die Bioverfügbarkeit von Vitamin B12 darüber hinaus reduzieren. Eine regelmäßige laborchemische Kontrolle und bei Bedarf eine Supplementierung von Vitamin B12 kann sinnvoll sein.

##### Zink

Zink ist als Co-Faktor der Superoxiddismutase im Radikalstoffwechsel von Bedeutung. Eine Supplementierung kann Störungen der Wundheilung positiv beeinflussen [43]. Die Evidenz für eine Supplementierung bei Menschen mit Diabetes ist als unzureichend anzusehen [44, 45].

##### Vitamin D

Ein Vitamin-D-Mangel steht im Zusammenhang mit Veränderungen des Glukose-metabolismus und der Insulinsekretion, jedoch ist die Evidenz für eine Vitamin-D-Supplementierung basierend auf systematischen Reviews und Metaanalysen als widersprüchlich anzusehen [46–48]. Bei Menschen mit T2DM und einem nachweisbaren Vitamin-D-Mangel kann es durch Supplementation zu einer Verbesserung der Insulinresistenz kommen [48].

##### Chrom

Eine nicht ausreichende Chromzufuhr wird mit einer gestörten Glukosetoleranz in Verbindung gebracht. Ein systematischer Review mit Metaanalyse von RCTs zeigte eine signifikante Reduktion der Nüchternblutglukose- sowie Insulinkonzentration, des HbA_1c_-Werts und des HOMA-IR [49], ein anderer Review konnte allerdings nur eine Reduktion des HbA_1c_-Wertes darstellen [50]. Es sind weitere Untersuchungen für eine optimale Supplementierung notwendig.

##### Selen

Der Selenstatus wird im Zusammenhang mit einem Diabetesrisiko und einem möglichen positiven Einfluss auf die glykämische Kontrolle von Personen mit Diabetes diskutiert. Sowohl ein niedriger als auch ein hoher Selen-Plasmaspiegel scheinen sich ungünstig auszuwirken (U-förmiger Zusammenhang). In einem systematischen Review wird festgehalten, dass die Evidenz für eine routinemäßige Supplementierung von Selen unzureichend ist [51].

##### Kalzium und Magnesium

Bei älteren Patient:innen mit T2DM, vor allem mit niedrigem BMI, wurde eine höhere Inzidenz für Schenkelhalsfrakturen gefunden [52]. Eine optimale Kalziumresorption ist nur bei gleichzeitig verfügbarem Vitamin D erreichbar. Es gibt Hinweise, dass eine Supplementierung mit Kalzium und Vitamin D mit einem geringeren Risiko eines T2DM verbunden ist. Allerdings muss die Evidenz dafür noch als unzureichend angesehen werden.

Ein unzureichender Magnesiumstatus wird mit einer schlechten glykämischen Kontrolle bei gestörter Glukosetoleranz [53, 54] und mit T2DM und assoziierten Komorbiditäten [55–57] in Zusammenhang gebracht. Die ausreichende Magnesium-aufnahme scheint die Progression einer eingeschränkten Glukosetoleranz zu T2DM zu verzögern [58, 59]. Die Evidenz für eine Magnesiumsupplementierung bei Menschen mit Typ 2 Diabetes ist allerdings unzureichend sowie widersprüchlich.

##### Antioxidantien

Da Diabetes mit erhöhtem oxidativem Stress verbunden ist, erscheint es möglich, dass bei schlecht kontrolliertem diabetischem Stoffwechsel der Bedarf an Antioxidantien erhöht ist. In verschiedenen Studien wurde eine inverse Beziehung zwischen der Antioxidantienzufuhr und dem KHK-Risiko gefunden [60, 61]. Die deutlichste Beziehung bestand für Tocopherole und β‑Karotin, der Effekt der Ascorbinsäure war geringer ausgeprägt. Klinische Studien, die den Effekt einer Tocopherol-Supplementierung in der Sekundärprävention der KHK untersuchten, kamen zu widersprüchlichen Ergebnissen. Die Supplementierung mit β‑Karotin zeigte keinen positiven Effekt, bei Rauchern wurde sogar ein höheres Krebsrisiko attestiert. Eine Supplementierung mit Antioxidantien kann derzeit aufgrund ungeklärter Effektivität und unbekannten Langzeitfolgen nicht empfohlen werden [61].

#### 1.5.2 Pflanzliche Nahrungsergänzungsmittel

Die Verwendung von komplementären und alternativmedizinischen Produkten wird von vielen Patient:innen gewünscht. Zimt ist eine der von Personen mit Diabetes am häufigsten verwendeten pflanzlichen Nahrungsergänzungen [62]. Zimt werden antioxidative, antiinflammatorische und antibakterielle Eigenschaften zugeschrieben. Bisher sind mehr als 200 Zimtarten bekannt [63]. Diese unterscheiden sich in der Zusammensetzung ihrer Inhaltsstoffe zum Teil signifikant. Dies und die Abhängigkeit des Gehalts der Inhaltsstoffe von Klima, Wetter, Bodenbeschaffenheit und Variationen in der Herstellung machen eine Standardisierung (d. h. immer gleicher Gehalt der Wirksubstanz) von Zimt schwierig. Die Verwendung größerer Mengen von Zimt, welcher zum Würzen beim Kochen und Backen verwendet wird, kann daher nicht zielführend sein.

Die Wirkung von Zimt auf die glykämische Kontrolle wurde bisher in unterschiedlicher Dosierung an kleinen Studienpopulationen mit unterschiedlicher Dauer (40 Tage bis 4 Monate) untersucht. Die Ergebnisse sind entsprechend inhomogen. Die Evidenz für eine Empfehlung der Supplementierung mit Zimt reicht nicht aus [63].

Die im Tierversuch gefundenen toxischen Effekte auf die Nierenfunktion werden kontrovers diskutiert [63]. Cassia-Zimt enthält darüber hinaus Cumarin, weshalb sich eine längerdauernde Einnahme auf die Blutgerinnung auswirken kann. Ceylon-Zimt hingegen enthält geringere Mengen Cumarin [63].

## 2 Lebensmittelbezogene Empfehlungen

### 2.1 Getreideprodukte/Vollkorn

Eine Ernährung reich an wenig verarbeiteten Vollkornprodukten, Hülsenfrüchten, Salat und Gemüse kann eine normo- und hypokalorische Energieaufnahme unterstützen und zu einer Gewichtsreduktion beitragen. Durch den reichlichen Verzehr von Lebensmitteln mit einer hohen Nährstoffdichte, also Lebensmittel, die einen hohen Gehalt an Nährstoffen in Relation zu ihrem Energiegehalt haben, wird eine gesteigerte Zufuhr gesundheitsfördernder Inhaltsstoffe wie Ballaststoffe, Mikronährstoffe und sekundäre Pflanzenstoffe bei gleichzeitig geringer Zufuhr stark verarbeiteter Nahrungsmittel mit gesundheitlich nachteiligen Inhaltsstoffen erreicht. Eine hohe Ballaststoffzufuhr wirkt sich positiv auf die Verdauung und Darmgesundheit aus und reduziert das Risiko für kardiovaskuläre Erkrankungen [10, 64–66].

In der Ernährungstherapie des Diabetes ist der Verarbeitungsgrad von Vollkornprodukten von Bedeutung. Jenkins et al. belegte bereits 1988 unterschiedliche postprandiale Auswirkungen von Vollkornbroten mit verschiedenem Gehalt an Vollkornmehl und grobkörnigem Getreide. Ein hoher Anteil an ganzen Getreidekörnern führt zu geringeren Blutglukosewerten. Die Samen- und Fruchtschalen des Getreides stellen für die Amylase eine physikalische Barriere dar und verlangsamen den Verdauungs- und Stoffwechselprozess. Vollkornprodukte mit einem hohen Anteil an ganzen Körnern bewirken einen geringen postprandialen Glukoseanstieg und sind für Menschen mit Diabetes ein wesentlicher, nicht-medikamentöser Therapiebestandteil [67].

Beim Konsum kohlenhydratbetonter Nahrungsmittel sollen Menschen mit Diabetes die tatsächliche Verzehrmenge und den Ballaststoffgehalt der Nahrungsmittel beachten, um die glykämische Reaktion autonom und positiv zu beeinflussen. Stark verarbeitete Vollkornprodukte und Getreideerzeugnisse aus fein vermahlenem Vollkorn sind für einen stabilen Glukoseverlauf begrenzt geeignet, da sie einen raschen postprandialen Blutglukoseanstieg bewirken können. Hierbei ist die Kombination dieser Nahrungsmittel mit weiteren Mahlzeitenkomponenten wie Proteinen und Fetten bzw. Ballaststoffen ausschlaggebend.

### 2.2 Obst, Gemüse und Hülsenfrüchte

Eine Ernährung, die vor allem reich an Gemüse ist, kann eine Gewichtsreduktion sowie die Versorgung mit Mikronährstoffen unterstützen. Während es bei Gemüse und Salat keine einschränkenden Verzehrempfehlungen gibt, können sich größere Mengen Obst oder Fruchtsäfte negativ auf die Blutzuckerkontrolle auswirken.

Die Weltgesundheitsorganisation (WHO) empfiehlt zur Prävention von T2DM täglich mindestens 400 g bzw. 5 Portionen Gemüse und/oder Obst, vorzugsweise aus frischem regionalem und saisonalem Angebot, zu verzehren [68]. Zudem erweist sich der Konsum von 100 g Leguminosen/Sojaprodukten aus ökologischen und sozialen Gründen laut Empfehlungen der EAT Lancet Commission als positiv. Als empfehlenswerte Mengen haben sich in der Praxis maximal zwei Handvoll Obst pro Tag und mindestens drei Handvoll Gemüse und Salat pro Tag erwiesen [69].

Hülsenfrüchte sollten vermehrt als Fleischersatz gegessen werden und wirken sich durch ihren Anteil an löslichen Ballaststoffen und resistenter Stärke positiv auf Blutzucker‑, Lipid- und Insulinspiegel aus [70, 71]. In einer randomisierten kontrollierten Vergleichsstudie senkten Leguminosen den HbA_1c_ stärker als eine Vollkornbasierte Ernährung [67]. Die Blutzuckerwirksamkeit von Hülsenfrüchten muss individuell ausgetestet werden und eine Berücksichtigung größerer Mengen im Rahmen einer prandialen Insulintherapie kann notwendig sein [70, 71].

Die empfohlenen Mengen Obst stellen eine wichtige Quelle für die Zufuhr an Vitaminen, Mineralstoffen, Spurenelementen und Ballaststoffen dar und machen Obst zu einer relevanten Lebensmittelgruppe in der ausgewogenen Ernährung von Menschen mit Diabetes. Durch gezielte Kombinationen von Obst, mit beispielsweise proteinreichen Nahrungsmitteln, wie einem naturbelassenen Milchprodukt, kann der Blutglukoseverlauf günstig beeinflusst werden [72–74].

Patient:innen mit prandialer Insulintherapie sollen beim Verzehr von Obst die entsprechenden KE berücksichtigen und die Insulinapplikation anpassen. Der Kohlenhydratgehalt von stärkereichen Gemüsesorten wie zum Beispiel Kartoffeln, Süßkartoffeln oder größere Mengen Kürbis muss ebenfalls mit Insulin abgedeckt werden.

Energiereiche und nährstoffarme Lebensmittel durch die empfohlene Menge an frischem Obst zu ersetzen, kann eine Gewichtsreduktion unterstützen [67, 75]. Der Konsum großer Mengen an Früchten und zuckerreichen Obsterzeugnissen wie Fruchtsäfte, Smoothies, zuckerreichen Convenience-Produkten, Trockenfrüchten soll vermieden werden, um unerwünschte Blutglukose-Reaktionen zu verhindern (klinische Erfahrung).

### 2.3 Getränkeauswahl

Um Empfehlungen zur Getränkeauswahl bei Diabetes evidenzbasiert aussprechen zu können, werden basierend auf aktuellen Kohortenstudien und randomisierten kontrollierten Studien die Daten als unzureichend bewertet [76]. Dennoch gilt eine Reduktion der Zufuhr von zugesetztem Zucker als positiv, da sie zu einer ausgewogeneren Ernährung beiträgt und die Kaloriendichte reduziert werden kann [77]. Um Blutglukosespitzen zu vermeiden, sollten Menschen mit Diabetes motiviert werden, ausschließlich kohlenhydratfreie Getränke, wie Wasser, Mineralwasser und ungesüßten Tee, zu sich zu nehmen. Fruchtsaftgetränke und Softdrinks sind aufgrund ihres hohen Zuckergehalts nur zur Bekämpfung einer Hypoglykämie geeignet. Süßstoffhaltige Light-Limonaden können in Maßen konsumiert werden. Light-Limonaden haben nach heutigem Erkenntnisstand keinen Einfluss auf metabolische Parameter wie Insulin, C‑Peptid, GLP 1, GIP, PYY, Glukagon oder HbA_1c_, [78–80]. Durch den süßen Geschmack reduziert sich jedoch bei vermehrtem Konsum die individuelle Süßschwelle und führt häufig zu größerem Verlangen nach süßen Getränken und Lebensmitteln.

#### Alkoholkonsum

Menschen mit Diabetes sollten über die Risiken und Auswirkungen von Alkohol-konsum auf den Blutzuckerspiegel sowie auf eine mögliche Verschlechterung der Stoffwechseleinstellung aufgeklärt werden. Durch die Beeinträchtigung der Gegen-regulationsmechanismen geht mit dem Genuss von Alkohol ein erhöhtes Risiko für v. a. nächtliche Hypoglykämien einher. Besonders zuckerreiche alkoholische Getränke, wie Bier oder Mischgetränke, können außerdem zu einem erhöhten Blutglukosespiegel führen. Als akzeptable Grenzwerte für den maßvollen Alkoholkonsum gelten für die Allgemeinbevölkerung sowie auch für Menschen mit Diabetes laut WHO 10 g Alkohol für Frauen und 20 g Alkohol für Männer pro Tag. Dies gilt auch für Menschen mit Diabetes. 10–12 g Alkohol entsprechen 0,3 l Bier, 0,125 l Wein oder 0,1 l Sekt. Weitere Details s. Leitlinie „Rauchen, erhitzte Tabakprodukte, Alkohol und Diabetes mellitus“.

### 2.4 Milchprodukte

Milch und Milchprodukte sind aufgrund ihrer Nährstoffdichte und ihres hohen Proteingehalts ein wichtiger Bestandteil der gesunden Ernährung bei Diabetes. Es ist dabei nicht notwendig, auf fettarme Milchprodukte zurückzugreifen [81].

Die aktuelle Datenlage zeigt positive Effekte von Milchfett bei T2DM sowie eine kardioprotektive Wirkung [82]. In Interventionsstudien wurde festgestellt, dass ein vermehrter Verzehr von Milchprodukten die Insulinresistenz verbessert. Fermentierte Milchprodukte wie Joghurt können das Risiko für T2DM ebenfalls senken [81]. Zudem wird eine positive Auswirkung von Milchsäurebakterien auf das Mikrobiom vermutet [82, 83]. Aktuelle Studien zeigen einen protektiven Effekt von vollfetten Milchprodukten auf das metabolische Syndrom auf [84]. Es konnte kein Zusammenhang zwischen gesättigten Fettsäuren aus Milch und einem höheren Risiko für KHK nachgewiesen werden [85].

Käse, als Vorspeise gegessen, erhöht die Magenverweildauer der Speisen und führt zu einem geringeren Blutzuckeranstieg [73]. Trotz des Gehalts an gesättigten Fettsäuren und der Kalorien konnte bisher kein Nachteil von Käsekonsum auf das Diabetesrisiko festgestellt werden [81, 86].

Aktuelle Studien zeigen keinen Zusammenhang zwischen Butterkonsum und KHK, jedoch eine protektive Wirkung auf Diabetes [87]. Butter sollte aufgrund des Fettgehalts trotzdem nur in Maßen gegessen werden.

Molkenprotein hat blutzuckersenkende Effekte und trägt zu einer langanhaltenden Sättigung bei. Zusätzlich stimuliert es die Muskelproteinsynthese stärker als andere Proteinquellen [88].

Alternative „Milchprodukte“ gehören hinsichtlich ihrer Zutatenzusammensetzung und Nährstofftabelle genauer betrachtet. Es sind Varianten ohne Zuckerzusatz zu bevorzugen. Jene Alternativen aus Getreidebasis weisen außerdem einen höheren Kohlenhydratgehalt auf.

Da Milchzucker kaum eine blutzuckererhöhende Wirkung aufweist, sollten Milchprodukte ohne Zuckerzusatz bevorzugt werden [83]. Fruchtjoghurts sind meist zuckerreich, alternativ empfiehlt sich stattdessen Naturjoghurt mit etwas Obst oder einem kleinen Löffel Marmelade.

### 2.5 Fleisch

Mageres Fleisch ist eine gute Proteinquelle und liefert Vitamine sowie Mineralstoffe. Verarbeitete Fleisch- und Wurstwaren sollten aufgrund ihres hohen Gehalts an Fett, Natrium, Nitrit, Zusatzstoffen und Hämeisen möglichst vermieden werden [89]. Rotes Fleisch und Wurstwaren durch andere Proteinquellen zu ersetzen, wirkt sich günstig auf Entzündungsprozesse und den Glukosestoffwechsel aus und senkt das Risiko für Krebs [90]. In Beobachtungsstudien steht eine fleischbetonte Ernährung in Verbindung mit einer erhöhten kardiovaskulären Mortalität [91]. Zudem besteht vor allem mit verarbeitetem rotem Fleisch eine moderate Beziehung mit Krebserkrankungen, KHK sowie T2DM [92]. Interventionsstudien zeigen eine Verbesserung zahlreicher metabolischer Parameter, wenn die täglich aufgenommene Fleischmenge reduziert wird [93–95]. Eine hohe Aufnahme von Hämeisen steigert das Risiko für KHK und begünstigt eine Insulinresistenz. Hämeisen erschwert zudem die Glukoseaufnahme in den Muskel. Leguminosen, Hühnerfleisch, Eier, Fisch, Milchprodukte, Nüsse und Vollkorngetreide sollten als Eiweißquelle bevorzugt werden [96].

### 2.6 Fisch

Für Menschen mit T2DM können die allgemeingültigen Empfehlungen der Österreichischen und Deutschen Gesellschaft für Ernährung, 1 bis 2 Portionen Fisch pro Woche zu verzehren, herangezogen werden.

Der regelmäßige Verzehr von Fisch, insbesondere von fettreichem Fisch (z. B. Lachs, Hering, Makrele), kann das Lipoproteinprofil im Blut positiv beeinflussen und das Risiko für die KHK-Mortalität und den ischämischen Schlaganfall herabsetzen [97]. Die positive Wirkung wird laut wissenschaftlicher Datenlage v. a. durch die Aufnahme der langkettigen Omega-3-Fettsäuren Eicosapentaensäure (EPA) und Docosahexaensäure (DHA) erreicht, wobei hier eine Aufnahme von 250 mg EPA und DHA pro Tag genügt, um die durch koronare Herzkrankheit bedingten Todesfälle vorzubeugen [98]. Diese Menge lässt sich durch den Verzehr von

1–2 Fischmahlzeiten pro Woche, wenn davon 70 g fettreicher Fisch verzehrt werden, abdecken. Allerdings variieren die Gehalte an EPA und DHA je nach Fischart, Fanggebiet, Nahrung oder Fütterung und Zubereitungsart, weshalb die Verzehrmenge von 70 g nur als Orientierungswert angesehen werden kann [99–101]. Eine ausreichende Evidenz, zur Empfehlung eines höheren Fischverzehrs (> 1–2 Portionen/Woche) bei Menschen mit T2DM sowie zur Supplementation langkettiger Omega-3-Fettsäuren (DHA und EPA), liegt nicht vor [26, 102]. Ebenso ist ein deutlich höherer Fischverzehr aus Gründen der Nachhaltigkeit und des Risikos einer zu hohen Aufnahme an unerwünschten Schadstoffen im Fisch (z. B. Methylquecksilber) nicht empfehlenswert. Dennoch herrscht nach Angaben wissenschaftlicher Literatur Einigkeit darüber, dass bei einem Fischverzehr von 1–2 Portionen pro Woche der gesundheitliche Nutzen durch Fischverzehr überwiegt [98, 103]. Beim Kauf von Fisch sollte auf eine nachhaltige Herkunft geachtet werden. Fisch aus Wildfang bietet gegenüber solchem aus Aquakulturen einen ernährungsphysiologischen Vorteil, da eine bessere Fettsäurenzusammensetzung vorliegt [104]. Für den Verzehr von Fisch an sich kann aus Sicht der Datenlage keine signifikante Assoziation hinsichtlich des Risikos für T2DM festgestellt werden [105]. Im Gegensatz dazu werden gesamte Ernährungsmuster, die Fisch einschließen (z. B. Mediterrane Ernährung), mit einem geringeren Risiko für Diabetes in Verbindung gebracht [106].

Abgesehen von den enthaltenen Omega-3-Fettsäuren EPA und DHA gibt es zahlreiche weitere Vorteile von Fischverzehr. Fisch ist ein nährstoffreiches Lebensmittel, das u. a. eine gute Quelle für Vitamin D, Jod, Selen und hochwertiges Protein darstellt [97].

### 2.7 Zucker und Süßungsmittel

Die WHO empfiehlt maximal 10 % der Gesamtenergiezufuhr aus zugesetztem Zucker zuzuführen. Für Menschen mit Diabetes gibt es keine eigenen Empfehlungen. Da Saccharose als Einfachzucker in isolierter Form, z. B. in Getränken, einen starken Blutglukoseanstieg verursacht, sollten Patient:innen motiviert werden, reinen Zucker weitgehend zu vermeiden und wenn nötig durch Alternativen zu ersetzen, die keinen Einfluss auf den Blutglukoseverlauf haben [107]. Eine Reduktion der Aufnahme von Mono- und Disacchariden in verarbeiteten Lebensmitteln und „Getränken“ erleichtert das Erreichen einer ausgeglichenen bzw. negativen Energiebilanz und damit die Gewichtsstabilisierung bzw. eine Gewichtsreduktion sowie eine Reduktion des Risikos für kardiovaskuläre Erkrankungen und der Entstehung einer Fettleber [2, 108].

Eine Ernährungsweise mit einem hohen Anteil an Haushaltszucker (> 20 % der Gesamt-Tagesenergie) führt sowohl bei Menschen ohne Diabetes als auch bei Personen mit Metabolischem Syndrom zu erhöhten Plasma-Triglyzeriden [109]. Die Reaktion der Triglyzeride auf Nahrungszucker ist abhängig von der aufgenommenen Menge und dem gleichzeitigen Konsum anderer Lebensmittel. Dem Zuckerkonsum von Patient:innen mit Metabolischem Syndrom (hohe Plasma‑, Triglyzerid-, niedrige HDL-Cholesterinspiegel) muss besondere Aufmerksamkeit gewidmet werden.

Süßstoffe können das Erreichen einer negativen Energiebilanz unterstützen [110]. Nach derzeitigem Wissen sind sie unter Einhaltung des ADI-Werts („Acceptable Daily Intake“) unbedenklich. Ein möglicher negativer Einfluss auf das Mikrobiom und die Glukosetoleranz wird diskutiert [111]. Es konnte kein Zusammenhang auf die Konzentration von GLP‑1, Peptid YY, Ghrelin und Glucose-dependent Insulinotropic Polypeptid (GIP), Glukagon sowie HbA_1c_ gezeigt werden [78, 80, 112]. Somit dürfte der Konsum von Süßstoffen keinen negativen Einfluss auf die Glukose- und Insulinregulierung bei T2DM haben. Für eine endgültige Aussage bedarf es jedoch weiterer Forschung und Langzeitstudien. Es ist jedenfalls darauf zu achten, dass die kalorische Einsparung durch die Verwendung von nicht-kalorischen Süßstoffen und Süßungsmitteln nicht über andere Lebensmittel oder Getränke kompensiert wird.

## 3 Mahlzeitenfrequenz

Mehr als drei Mahlzeiten pro Tag können oftmals einen Mitgrund einer Gewichtszunahme darstellen. Dies scheint durch eine insgesamt erhöhte Energie-aufnahme und den durch häufige Mahlzeiten verursachten erhöhten Insulinspiegel verursacht zu werden [113]. Zudem wird durch häufige Zwischenmahlzeiten das natürliche Hungersignal unterdrückt.

Während frühere Diabetestherapien aufgrund des hohen Hypoglykämierisikos Zwischenmahlzeiten oft notwendig machten, ist es mit den heutigen modernen Therapiemöglichkeiten zuallermeist nicht mehr notwendig, Zwischenmahlzeiten zu essen. Die Entscheidung, welche Mahlzeitenfrequenz für welchen Menschen mit Diabetes optimal ist, sollte individuell, angepasst an die persönlichen Bedürfnisse und die Diabetestherapie, getroffen werden. Zur Gewichtsreduktion bzw. -stabilisierung und für gleichmäßige Blutglukoseverläufe empfiehlt es sich meist ein 3‑Mahlzeiten-Prinzip einzuhalten. Intermittierendes Fasten wird im Abschn. 4.3 näher diskutiert.

## 4 Ernährungsformen und Gewichtsreduktion

### 4.1 Low Carb/Low Fat

Eine generelle Reduktion der Kohlenhydrataufnahme zur Verbesserung der Stoffwechsellage wird immer wieder diskutiert. Diese Reduktion wird üblicherweise dann unter dem Terminus „Low Carb Diät“ subsummiert. Der Ausdruck „low-carb“ ist eigentlich falsch – es müsste „low carb high fat“ (LCHF) Diät genannt werden [114]. Nach derzeitiger Definition spricht man von einer LCHF-Diät, wenn 50–150 g Kohlenhydrate pro Tag verzehrt werden. Eine Kohlenhydratreduktion wird je nach Intensität in very-low-carb, low-carb und moderate-carb klassifiziert. Letzteres wird mit 130–230 g (26–45 E%) definiert [115]. Eine ketogene Diät, die Extremform der LCHF-Diät, erlaubt einen Kohlenhydratverzehr von 20–50 g pro Tag [116, 117]. Ziel der LCHF-Ernährung bzw. ihrer Extremform, der ketogenen Ernährung, ist, dass durch die Kohlenhydratreduktion weniger Glukose als Energielieferant zur Verfügung steht, der Insulinspiegel sinkt und der Körper durch Lipolyse Energie gewinnt, dies führt auf längere Sicht zu Exsikkose. Nach dieser Hypothese müsste es einen Wert geben, ab dem diese metabolischen Veränderungen auftreten. Eine Kohlenhydratreduktion auf unter 45 % der aufgenommenen Energie kann zu Therapiebeginn mit einer stärkeren Reduktion des HbA1c assoziiert sein. Langfristig ist sie einer Diät mit einem höheren Kohlenhydratanteil nicht überlegen [4, 118].

Zum derzeitigen Zeitpunkt fehlen gute Vergleichsstudien, ob eine LCHF-Ernährung einer „low fat, high carb“-Ernährung bei Menschen mit Diabetes wirklich zu bevorzugen ist. Eine Metaanalyse zeigte, dass zumindest über einen kurzen Zeitraum eine LCHF-Diät zu einer Verbesserung der Blutzuckereinstellung und zu einer Gewichtsabnahme bei Personen mit T2DM führt [119]. Eine aktuelle Metaanlyse zeigte, dass low-carb (< 130 g KH/Tag) gegenüber low-fat nach sechs Monaten zu einer höheren Diabetesremission führen könnte, indem signifikant mehr Patienten einen HbA_1c_ unter 6,5 % erreichten. Allerdings waren die Unterschiede bei Remission < 6,5 % ohne Medikation und längerer Intervention nicht mehr signifikant. Die Verbesserung von Triglyceriden und Insulinsensitivität sowie Gewichtsverlust waren vor allem nach 6 Monaten zu beobachten, die sich jedoch nach 12 Monaten verringerten [120, 121]. Eine „very-low-carb“-Diät (< 10 E%) erreichte nach 6 Monaten eine weniger wirksame Gewichtsreduktion als eine low-carb-Diät. Das lässt sich wiederum durch die mangelnde Adhärenz dieser Ernährungsform erklären. Nach sechs Monaten erreichten die Patienten keinen signifikanten Unterschied in der Lebensqualität, jedoch nach 12 Monaten eine klinisch bedeutsame, aber statistisch nicht signifikante Verschlechterung der Lebensqualität [120, 122].

Noch deutlicher sind diese Resultate bei einer ketogenen Diät sichtbar. Allerdings, wie bei allen extremen Ernährungsformen, ist die Therapieadhärenz eingeschränkt. Da der Verzehr von Kohlenhydraten noch dazu eine angenehme hedonische Wirkung hat, kann eine LCHF-Diät mit verringertem Genuss und Freude verbunden sein. Das wiederum könnte der Grund sein, warum eine längerfristige Einhaltung dieser Diät ein Problem darstellt [123]. Darüber hinaus ist zu bedenken, dass die meisten Patient:innen eine Kohlenhydratreduktion durch eine höhere Fettaufnahme kompensieren. Bei Nichtbeachtung der Qualität der Kohlenhydrate und Fette könnte das langfristige Risiko für kardiovaskuläre Erkrankungen steigen. Weiters kann diese Einschränkung der Lebensmittelauswahl mit einem Risiko einer unzureichenden Nährstoffversorgung verbunden sein sowie sich negativ auf die Aufnahme von Ballaststoffen auswirken [123, 124].

Pauschal gesehen dürfte also immer noch die Gesamtkalorienaufnahme der beste Prädiktor für Gewichtsverlust und Verbesserung der glykämischen Stoffwechsellage sein und nicht eine alleinige Reduktion der Kohlenhydrate [114]. Eine kohlenhydratarme Ernährung kann für Personen mit Diabetes und Übergewicht sowie Adipositas eine kurzwirksame (bis zu 6 Monaten) Möglichkeit zur Verbesserung der glykämischen Kontrolle und der Triglyceride darstellen und sollte unter medizinischer und diätologischer Begleitung erfolgen. Einerseits gilt es zu beachten, dass bei einer Umstellung auf eine kohlenhydratärmere Ernährung das Hypoglykämie-Risiko steigt und somit bei Bedarf die Diabetes-Medikation angepasst werden muss, andererseits ist es durch die Einschränkung der Lebensmittelauswahl besonders von Bedeutung ernährungstherapeutisch zu unterstützen, um eine ausreichende Ballaststoffzufuhr in Form von Vollkornprodukten und Obst sowie Gemüse sicherzustellen und die Zufuhr von gesättigten Fettsäuren gering zu halten [122].

### 4.2 Mediterrane Ernährung

Die traditionelle mediterrane Ernährung ist ebenfalls als kohlenhydratreduzierte Ernährungsform einzustufen, welche von der ADA und EASD verglichen zu low-carb als übergeordnet eingestuft wird [125]. Der in der Literatur beschriebene Terminus „Mediterrane Diät“ impliziert durch das Wort Diät einen streng abgestimmten Mahlzeitenplan, vielmehr sollte es als eine Art Lebensweise verstanden werden, bei der der Genuss von saisonalen und frischen Mahlzeiten im Vordergrund steht, und die den Verzehr von mehr Gemüse, Hülsenfrüchten, Nüssen, Samen, frischem Obst, vollkornreichen Lebensmittel, Olivenöl und Fisch sowie moderaten Konsum von Joghurt, Käse und Eiern und wenig rotem Fleisch vorsieht. Nach derzeitigem Stand der Wissenschaft zeigt die traditionelle mediterrane Ernährungsweise die besseren Erfolge bezüglich der Nüchternblutglukose und des Lipidprofils. Die positiven Auswirkungen auf Gewichtsreduktion, HbA_1c_ und Blutdruck lassen die mediterrane Ernährung neben „low-fat“ und „low-carb“ zu den drei idealen Ernährungsweisen zählen [126–128]. Weitere Untersuchungen werden benötigt, um den Stellenwert dieser Ernährungsform in der Diabetestherapie zu erheben.

### 4.3 Intervallfasten

Das immer größer werdende Interesse für intermittierendes Fasten wirft auch die Frage auf, ob dies Vorteile für Menschen mit Diabetes bringen kann. Fasten bedeutet eine gewisse Zeitperiode auf Lebensmittel, Getränke oder beides zu verzichten und hat oftmals religiöse oder spirituelle Hintergründe. Beim Fasten gilt als Ansatzpunkt die Mahlzeitenhäufigkeit zur Gewichtsreduktion und Stoffwechselverbesserung und nicht die qualitative Optimierung der Ernährung. Je nach Art des Fastens werden Zeitangaben vorgegeben, wann Essen erlaubt ist und wann nicht, ohne Angabe von genauen Ernährungsempfehlungen. Dies lässt Fasten auf den ersten Blick unkompliziert und einfach erscheinen. Verbote gibt es meistens nicht und der Verzicht beschränkt sich immer nur auf ein paar Stunden oder Tage in der Woche – Wechselfasten, 5:2 Fasten oder zeitlich begrenztes Fasten, wie 16:8. Das mag die Compliance im Vergleich zu anderen Diäten erhöhen. Jedoch ist oft nicht klar, was am „Festtag“ alles verzehrt werden darf, da eine Ernährungsumstellung nicht im Fokus liegt. Das kann wiederum zu unkontrollierten Schlemmereien führen. Eine ausgewogene, kalorienreduzierte Ernährung wäre nach wie vor das Ziel einer Gewichtsreduktion.

Beobachtungsstudien im Rahmen des Ramadans sehen bei Gesunden nur geringe und vorübergehende metabolische Veränderungen [129–131]. Die Evidenz in Hinblick auf Vorteile einer geringeren Mahlzeitenfrequenz zugunsten einer Verbesserung auf Körpergewicht, Fettmasse und Taillenumfang ist gering [129].

In Metaanalysen zum Intervallfasten finden sich keine Vorteile des Intervallfastens gegenüber einer kontinuierlichen Kalorienrestriktion. Verglichen mit einer unveränderten Kontrolldiät kommt es zu einer signifikant höheren Reduktion von Körpergewicht, Taillenumfang, Blutdruck und Triglyceriden, nicht aber von LDL-Cholesterin, Nüchternglukose oder HbA1c [132, 133].

Kleinere randomisierte kontrollierte Studien mit Personen mit Diabetes konnten zeigen, dass intermittierendes Fasten im Vergleich zu Nicht-Fasten, entweder an aufeinanderfolgenden Tagen oder durch Fasten von 16 h oder mehr, zu einem Gewichtsverlust führen kann, allerdings zu keiner Verbesserung des HbA1cs [134]. Ein erhöhtes Hypoglykämierisiko sei zudem nicht außer Acht zu lassen. Generell liegt derzeit aber nur eine beschränkte Anzahl an qualitativ guter RCTs mit Personen mit Diabetes vor [135–137].

An den Fasttagen können zudem Nebenwirkungen auftreten, da es zu einem Abfall des Blutzuckerspiegels kommt. Mögliche Nebenwirkungen sind Schwindel, Schlaf-störung, Mundgeruch, Kopfschmerzen, Konzentrationsschwäche, vermehrtes Kälte-empfinden und Hungergefühl. Fasten ist für Kinder, Jugendliche, Schwangere, Stillende, Senioren mit Erkrankungen, Menschen mit Essstörungen oder chronisch Erkrankte nicht empfehlenswert.

### 4.4 Formuladiäten

Da 60–90 % der Personen mit T2DM übergewichtig bzw. adipös sind, stellt eine Gewichtsreduktion eine außerordentlich wichtige Säule in der Therapie dar. Formuladiäten gehen mit einer stark hypokalorischen Ernährung einher und fördern besonders bei übergewichtigen und adipösen Personen mit T2DM einen raschen Gewichtsverlust. Bei einer Forumladiät wird mindestens eine Mahlzeit am Tag durch ein kalorienreduziertes Produkt in Form von industriell hergestellten Shakes, Fertigdrinks etc. ersetzt. Dieser wiederum resultiert in den meisten Fällen in einer signifikanten Verbesserung des Glukose- und Fettstoffwechsels [138, 139].

Formuladiäten können als erste Unterstützung dienen und sollen nur unter medizinischer und ernährungstherapeutischer Begleitung zum Einsatz kommen. Ziel sollte aber eine langfristige Umstellung zu einem gesundheitsfördernden Ernährungsverhalten sein.

## 5 Ernährungsempfehlungen bei Diabetes Typ 1

Die Ernährungsempfehlungen bei Menschen mit T1DM unterscheiden sich nicht von jenen der gesunden Allgemeinbevölkerung. Das Ziel ist eine ausgewogene und bedarfsdeckende Ernährung unter Erreichung nahe-normoglykämischer Werte. Bei bestehendem Übergewicht empfiehlt sich eine hypokalorische Ernährung unter diätologischer Begleitung zur langfristigen und nachhaltigen Gewichtsreduktion [140, 141].

Menschen mit prandialer Insulintherapie im Rahmen einer funktionellen Insulintherapie (FIT-Therapie) bzw. Insulinpumpentherapie (kontinuierliche subkutane Insulin-Infusion [CSII]) wird für den Konsum kohlenhydrathaltiger Lebensmittel die Berechnung und in weiterer Folge Einschätzung des Gehalts an Kohlenhydraten in Gramm bzw. KE und der postprandialen Reaktion des Nahrungsmittels bzw. der Speise empfohlen. Aufgrund von technologischen Fortschritten und um eine länderübergreifende Vereinheitlichung zu ermöglichen, sollen neudiagnostizierte Patient:innen auf Gramm an Kohlenhydraten bzw. KE (1 KE = 10 g Kohlenhydrate) anstelle der bisher üblichen Broteinheiten geschult werden. Als unterstützende Instrumente für die Abschätzung des postprandialen Blutzuckerverlaufs eignen sich der GI und die „Kohlenhydrat-zu-Ballaststoff-Ratio“ (KH:Bst-Ratio).

Das Wissen über die postprandiale Wirkung kohlenhydrathaltiger Speisen und Getränke, das Berechnen und Einschätzen von KE bzw. der Kohlenhydratmengen in Gramm sind für die Bestimmung der prandialen Insulindosierung notwendig. Da das Austesten der individuell benötigten Kohlenhydratmenge im Vordergrund steht, kann keine explizite Mengenempfehlung ausgesprochen werden [27, 108, 140]. Das Berechnen und Einschätzen der Kohlenhydratmengen einer Mahlzeit soll in strukturierten Ernährungsschulungen durch spezialisierte Diaetolog:innen erlernt werden.

Für alle Patient:innen mit einem Insulinmangeldiabetes, insbesondere einem T1DM, ist eine ketogene Diät bzw. Very-Low-Carb-Ernährungsweise nicht zu empfehlen, da das Risiko einer Ketoazidose aufgrund einer zu drastischen Insulinreduktion nicht unterschätzt werden darf. Dies kann besonders riskant sein, wenn diese Patient:innen mit SGLT2-Hemmern behandelt werden. Es sind weitere Studien mit einer größeren Studienpopulation sowie einer längeren Dauer notwendig, um exakte Empfehlungen geben zu können.

Eine individuelle Berücksichtigung der Krankheit inklusive Begleit- und Folgeerkrankungen, der Präferenzen und des Lebensstils des Patienten sind notwendig, um eine geeignete Ernährungsform langfristig umsetzen zu können [2, 27, 108].

Im Update 2023 der Ernährungsempfehlungen für Diabetes mellitus wurden nun lebensmittelbezogene Ernährungsempfehlungen angeführt, um bessere praxisbezogene Empfehlungen abgeben zu können. Zudem werden Ernährungsformen wie, low-carb/low-fat, mediterrane Ernährung, Intervallfasten und Formuladiät, in eigenen Kapiteln diskutiert. Abschließend werden Ernährungsempfehlungen bei Diabetes Mellitus Typ 1 angeführt.

## Abkürzungen

ADI: „Acceptable daily intake“
ALA: Alpha-Linolensäure
BMI: Body Mass Index
CSII: Kontinuierliche subkutane Insulin-Infusion
DHA: Docosahexaensäure
DSMES: Diabetes-Selbstmanagement
E%: Energieprozent
EPA: Eicosapentaensäure
FIT-Therapie: Funktionelle Inuslintherapie
GI: Glykämischer Index
GL: Glykämische Last
HDL: High density Lipoprotein
KE: Kohlenhydrateinheiten
KHK: Koronare Herzerkrankung
KG: Körpergewicht
LCHF: Low carb, high fat
LDL: Low density Lipoprotein
PUFA: Mehrfach ungesättigte Fettsäuren
T1DM: Diabetes mellitus Typ 1
T2DM: Diabetes mellitus Typ 2
WHO: World Health Organization
